# Maintenance of Coastal Surface Blooms by Surface Temperature Stratification and Wind Drift

**DOI:** 10.1371/journal.pone.0058958

**Published:** 2013-04-11

**Authors:** Mary Carmen Ruiz-de la Torre, Helmut Maske, José Ochoa, César O. Almeda-Jauregui

**Affiliations:** Biological Oceanography Department, Centro de Investigación Científica y de Educación Superior de Ensenada, Ensenada, Baja California, Mexico; University of Vigo, Spain

## Abstract

Algae blooms are an increasingly recurrent phenomenon of potentially socio-economic impact in coastal waters globally and in the coastal upwelling region off northern Baja California, Mexico. In coastal upwelling areas the diurnal wind pattern is directed towards the coast during the day. We regularly found positive Near Surface Temperature Stratification (NSTS), the resulting density stratification is expected to reduce the frictional coupling of the surface layer from deeper waters and allow for its more efficient wind transport. We propose that the net transport of the top layer of approximately 2.7 kilometers per day towards the coast helps maintain surface blooms of slow growing dinoflagellate such as *Lingulodinium polyedrum*. We measured: near surface stratification with a free-rising CTD profiler, trajectories of drifter buoys with attached thermographs, wind speed and direction, velocity profiles via an Acoustic Doppler Current Profiler, Chlorophyll and cell concentration from water samples and vertical migration using sediment traps. The ADCP and drifter data agree and show noticeable current shear within the first meters of the surface where temperature stratification and high cell densities of *L. polyedrum* were found during the day. Drifters with 1m depth drogue moved towards the shore, whereas drifters at 3 and 5 m depth showed trajectories parallel or away from shore. A small part of the surface population migrated down to the sea floor during night thus reducing horizontal dispersion. The persistent transport of the surface bloom population towards shore should help maintain the bloom in favorable environmental conditions with high nutrients, but also increasing the potential socioeconomic impact of the blooms. The coast wise transport is not limited to blooms but includes all dissolved and particulate constituents in surface waters.

## Introduction

In the last decades, dense algal blooms, historically often referred to as red or brown tides, have been studied worldwide because of their socioeconomical and ecological implications [1], [2]. The increasing frequency of blooms has been related to coastal eutrophication [3], climatic shifts [4], and the transport of algal species by ship ballast water [5]. The spatial distribution of algal blooms is determined by the interaction between biological and ecological features of the organism on the one hand, and by physical oceanographic structures and processes on the other hand [6]. Despite a large number of publications related to coastal surface blooms, little is known about hydrographic conditions that promote the formation and maintenance of blooms [1], [7]. Here, we consider oceanographic aspects of surface bloom transport, but the processes we describe are relevant for all surface water constituents, such as wastewater, nutrients or harmful algal bloom forming species, all of these having potentially socioeconomic impacts [8].

**Figure 1:**
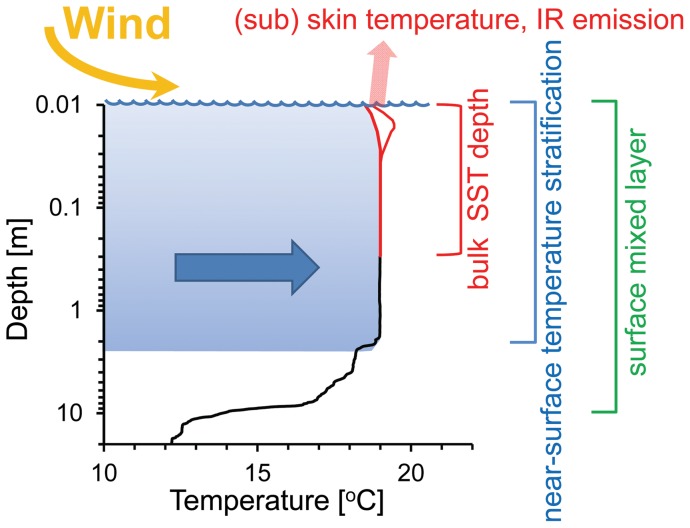
Schematic representation of different thermal stratification depth scales. Wind forcing (yellow arrow) induces the movement of the near-surface layer (big arrow within the water column).

Surface transport is determined by several factors, including coastal morphology, wind patterns and water column stratification. Thermal stratification is crucial in controlling the horizontal dynamics of the upper ocean at different scales [9]; temporal scales associated with surface water layers are days to weeks, and relevant spatial scales range from decimeters to meters in thickness [10]. Figure 1 compares the near surface temperature stratification (NSTS) with sea surface temperature (SST) and the seasonal thermocline; these different components are controlled largely by the same meteorological conditions but at different time scales [11]. High solar irradiance and low surface mixing rates promote stratification and the formation of a diurnal thermocline in clear ocean waters [12]; this thermocline, passes through different phases: the formation and the increasing of thickness during the day [13]. Note that the vertical scale of sea surface temperature is restricted to the first few centimeters and can be related to neuston organisms; here we are concerned with the more extensive vertical scale for surface blooms and NSTS (Figure 1). Vertical density structure defines the current shear pattern between the wind drift at the surface and the deeper water. With NSTS there can be significant difference in current bearings within few meter depth intervals, which can influence the distribution of phytoplankton [14]. In coastal upwelling areas, alongshore wind is a dominant factor inducing Ekman drift directed offshore. The thermal wind breeze often associated with coastal upwelling has a direct influence on the dynamics of the surface mixed layer [15] and on the onshore transport of surface waters [16], [17], [18]. Sea breezes occur at two-third of earth's coastlines [19] and near 30° latitude are expected to generate, pronounced, near inertial, oceanic motions [20]. This research was partially motivated by the recurrent formation of dense surface blooms in coastal waters off Baja California, lasting from weeks to months. The dominant surface bloom organisms that make up red tides are slow growing dinoflagellates. Under favorable growth conditions their generation time is about 2 days [21], which makes the persistence of these blooms more difficult to explain. Part of the explanation may relate to the dinoflagellates diel vertical migration that is supposed to increase survival and competitive success [22], [23].

Vertical migration involves geotaxis, a circadian rhythm and chemosensory behavior, the general pattern of these photosynthetic species is to move to shallower depths during the day and to deeper waters at night for nutrient acquisition and predator avoidance. The specifics of this behavior depend on the species and environmental conditions. The vertical migration can be about 16 m day^−1^ with vertical velocities up to 1–2 m h^−1^ or more (280–560 µm s^−1^) [24]. Typically vertical migration extends down to the pycnocline or nutricline [25], [26]; however, not all dinoflagellates show this pattern. For example, on the east coast of United States, *Karenia brevis* has been shown to swim directly toward the sediment to acquire organic and inorganic nutrients from pore water to alleviate bottom-up controls and permit populations to persist as vegetative cells near the sediment – water interface [27].

Our study was based on the assumption that diurnal near surface stratification together with the onshore wind contributes to the maintenance of surface blooms. NSTS was expected to reduce the frictional coupling with the lower water column thus facilitating the wind transport of the surface layer. The sea breezes in coastal upwelling areas such as the California current system are driven by the temperature gradient between the land and the sea and are directed towards the coast during daytime [28], [20]. Our results show that near surface stratification is a common occurrence during the day in our coastal waters but the form of stratification is different from our initial concept of a homogenous near surface layer (Figure 1). We could document the expected transport of the surface bloom that should help slow growing dinoflagellates such as *Lingulodinium polyedrum* to maintain high cells densities closer to the coast during a prolonged time in a region with coastal and tidal currents, facilitating the permanence of dense algal blooms. The wind transport of the surface layer will have to be considered in the horizontal distribution of any dissolved and particulate constituent in this layer.

## Materials and Methods

### Ethics statement

No specific permits were required for the described field studies. The location is not privately-owned or protected in any way and the field studies did not involve endangered or protected species.

### Study site and water samples

**Figure 2:**
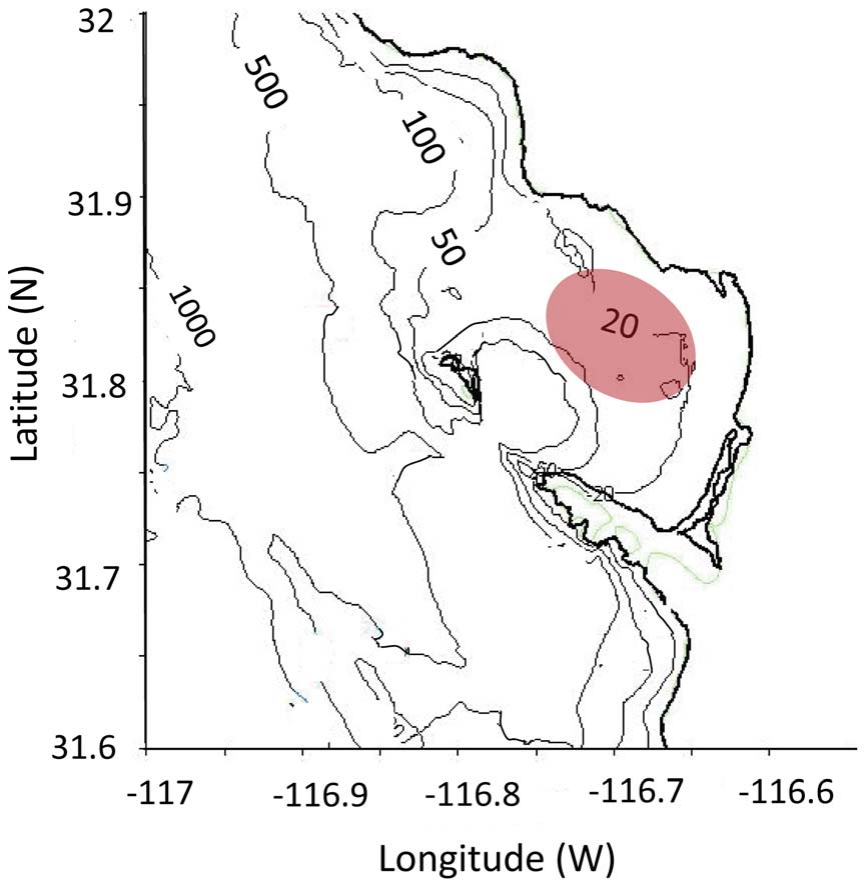
Study site, Todos Santos Bay. Baja California, Mexico (31° 40′ to 31° 56′N and 116° 36′ to 116° 50′W).

The fieldwork was carried out in Todos Santos Bay (TSB, 31° 40′ to 31° 56′N and 116°36′ to 116° 50′W) on the northwestern Baja California coast. TSB is an open coastal bay of approximately 10 km diameter and two small islands located near the southwestern corner of the entrance. Surface water characteristics within the TSB are closely related to the cold California Current [29]. Data for the present study were collected on October 4, 5, 6, 11, 12, and 18, 2011 during a *L. polyedrum* bloom. The sampling, profiling, and drifter deployment started at the time the bloom patches could be observed at the surface, about 10∶00 am and finished when surface patches disappear (around 17∶00 hrs.). Sampling locations were chosen depending on the detectability of patches (Figure 2). ADCP deployments used to compare the performance of drifters were during pre-bloom on September 21, 28 and 29, 2011. Sediment traps were deployed October 5, 11 and 18, 2011 at around 17∶00 hrs.

Water samples were taken with Niskin sampler within bloom patches at 1, 3, 5 m for chlorophyll and phytoplankton counts on October 4, 5, 6, 11, 12, and 18, 2011. Phytoplankton samples were preserved with Lugol and kept with chlorophyll samples in the dark with ice until return to the laboratory. Chlorophyll a was filtered on glass fiber filters (GFF, Whatman) with sample volumes from 0.01 to 0.5 l depending on sample concentration. The filtered samples were frozen at −40°C until extraction with 90% Acetone at −20°C for 21 hours and then measured with a fluorometer (Turner Design 10) according to Welschmeyer [30]. Phytoplankton was counted with a transmission microscope (Axioskope 2 plus) at 200X magnification.

### Near surface thermal stratification

**Figure 3:**
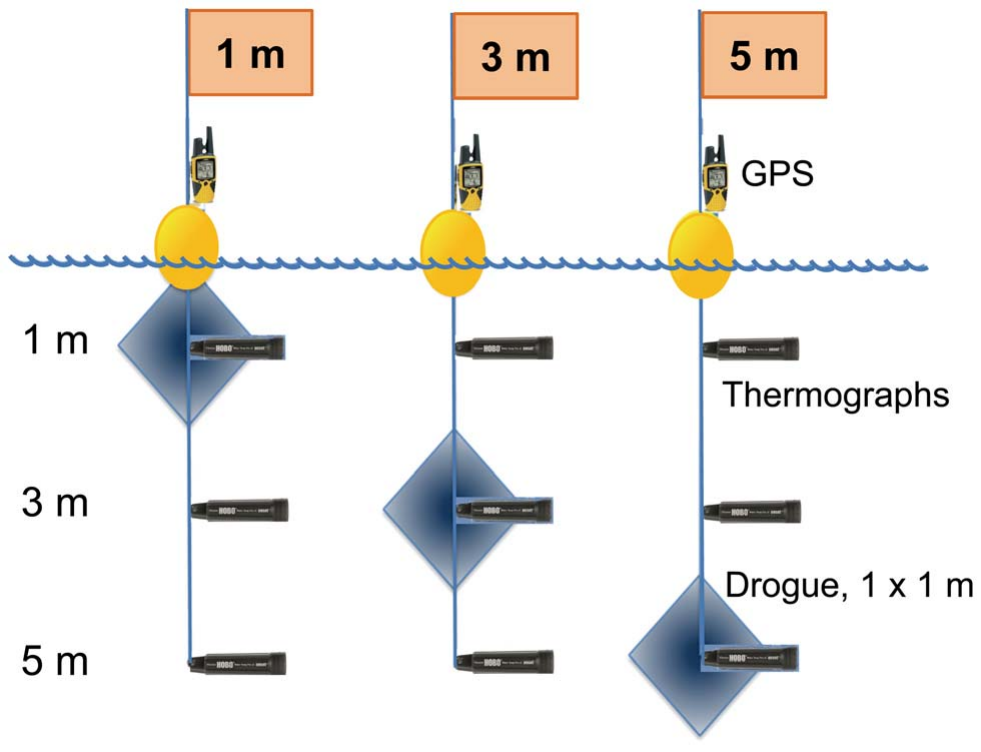
Schematic representations of CODE-type drifters. Each drifter consisted of flag, GPS attached above floatation (yellow ovals), kite-type drogues of 1 m^2^ in two directions (blue diamond), and 3 thermographs at depths 1, 3 and 5 m.

A free-rising CTD [3] was used to document the vertical structure of temperature in the water column up to 0.4 m below the surface with a nominal vertical resolution of 0.05 m. The profiler included sensors for temperature, conductivity, pressure (RBR), PAR (Biospherical), chlorophyll fluorescence and particle back scattering (Wetlabs). A comparison of the 0 to 5 m values of particle backscatter and chlorophyll fluorescence showed no near surface photoinhibition of the chlorophyll fluorescence (data not shown). The chlorophyll fluorometer data were calibrated against extracted chlorophyll concentration measured in concomitantly sampled water (R^2^ = 0.8476, p<0.05). A total of up to 15 vertical profiles per day were obtained during the 6 sampling days. To assess thermal stratification and to document the pattern of the formation of the superficial thermocline, three thermographs (Hobo U22 Pro V2) were attached at 1, 3 and 5 m to each CODE-type drifter (Figure 3). Thermographs recorded water temperature every 5 minutes during the sampling period with 0.01°C resolution and 0.2°C accuracy. The thermographs were completely covered with one layer of white electric tape to avoid solar heating of the black thermograph body and to protect the communication window. To assure instrument comparability, temperature measurements started at least one hour before the deployment with all thermographs kept together in an icebox. A non-parametric test (Kruskal-Wallis) was used to assess temperature difference among depths, and among drifters (Statistica v.7.1).

### Horizontal water movement

#### Drifter's deployments

To document the horizontal water movement, three CODE-type drifter buoys were deployed during the day. The projected drogue areas were 1 m^2^ placed at 1, 3, or 5 m depth. To document the drifter's trajectories, a GPS Rino 110 (Garmin) was attached to their surface buoy (Figure 3). GPS data were downloaded and processed using MapSource software version 6.16.3 (Garmin). The time and location of drifter deployment and recovery were registered. Two additional Holey Sock type drifters (1 m diameter, 1m length) positioned at 1 m and 5 m depth were deployed together with the Code type drifters at the same depth. Holey sock type drifters did not have a GPS attached, but deployment and recovery locations and times were registered to compare both drifter types. We found a good agreement between Holey Sock and Code type drifters either in velocities (R^2^ = 0.78627, p>0.05) and bearing (R^2^ = 0.9162, p>0.05), adding confidence to the performance of Code-type drifters. We used Matlab (v7.9, The MathWorks) to create an animation and visualize the motion path pattern behavior of the three Code type drifters and wind direction for October 5 and 6, 2011 (Video S1).

### ADCP and CODE type drifter comparison

Detailed comparisons of drifters' trajectories and ADCP velocities were done for three days, prior to the bloom. An Acoustic Doppler Current Profiler (ADCP) (Aquadopp Nortek, 1 MHz) was anchored, looking upward from 11 m depth and profiling close to the surface, in 21 layers, 0.5 m thick. In the upper meter the surface reflection is expected to produce noise that is reduced by time integration. Considering the magnetic declination, the West to East (u) and South to North (v) currents were inferred with the ADCP in 2 min averages. Drifter-derived velocity was compared with the velocity measured by the ADCP at 1, 3 and 5 m to evaluate their water following capability, or its degree of agreement with the ADCP measurements. Each drifter was deployed at approximately 250 meters distance from the anchored ADCP and the drifter position was registered, via a GPS, every 5 minutes for at least 2 hours. The ADCP data and trajectories were obtained during September 2011 on the 2nd (13∶30 to 15∶30 hrs), 21st (14∶10 to 15∶40 hrs) and 29th (11∶00 to 16∶00 hrs).

Data comparison included: i) time series of velocities, by inferring drifter velocities based on their motion path, ii) inferred trajectories, by integration of ADCP velocities over time. In the latter, the virtual trajectories project particle trajectories according to measured velocity by the moored ADCP. In the first comparison the measured velocities are at different locations. Both comparisons make the assumption that the lateral scale of velocity variability is larger than the separation of drifters and current meter. Drifter and ADCP measurements were compared during a non-bloom, low wind strength period, therefore these measurements are useful for comparisons but the data are not typical for bloom conditions.

### Current and wind pattern measurements during the bloom

For each CODE type drifter deployed during the bloom, we used the latitude (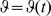) and longitude (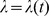) information, where 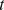 is time to calculate the West to East velocity, or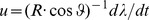, and the South to North velocity, or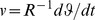, where 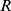
is earth's radius. The differentiation was approximated by centered finite differences using one sample before and another sample after the time of interest (i.e. 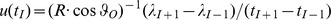 and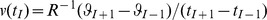, where the index 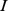
runs through the sequence of consecutive measurements, and 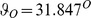, the latitude of the anchored ADCP). For *n* drifter positions recorded during a deployment, we computed n^−2^ velocity vectors.

Virtual trajectories that follow from the ADCP recordings were computed as


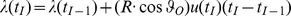
, and 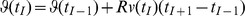.

To analyze the path of the drifters we plotted the drifter trajectories, after removal of outliers so the track was relatively smooth and continuous. We kept the part of the trajectories that coincided in time; removing all data prior to the entry of the last drifter into the water as well as all data after the first drifter was picked up. For the wind we use here the direction of propagation, opposed to the meteorological convention. Wind velocity and direction data from sampling period (04, 05, 06, 11, 12 and 18 October, 2011) were obtained from the meteorological station of the Naval Secretariat (SEMAR), located in its Oceanographic station near the Port of Ensenada Baja California. Concurrent observations of October average wind intensity and direction were obtained from a meteorological station, managed by the Centro de Investigación Científica y de Educación Superior de Ensenada (CICESE), and located on shore less than 4 km from our observation point. The average of wind direction and velocity and its standard deviations were plotted during local solar time. Wind speed and wind direction for each sampling day were processed for the intervals of sea breeze peaks (13–15 hrs.) to compare with the velocities and trajectories of 1 and 5 m drifters during the bloom.

### Assessment of vertical migration of *L. polyedrum*

Sedimentation of vegetative cells *L. polyedrum* cells was monitored by means of six sediment traps placed on the sea floor at approximately 14 m depth below surface bloom patches at about 16∶30 hrs on October 5, 11, and 18, 2011. Before deployment a mixture of chilled salt brine and HgCl_2_ was added to three traps, and chilled salt brine was added to the other three traps. The traps were collected around 9∶00 hrs, the next day. Each trap consisted of a PVC tube (42 cm height, 10.7 cm diameter) with four inside baffles to avoid resuspension of sample during recovery of the trap, the mouth of the trap was 0.55 m about the sea floor. Samples were kept dark and cold until arrival at the laboratory. Given the lack of statistically significant difference between traps with and without HgCl_2_, we used the average of cell abundance in the six traps to calculate sedimentation.

**Figure 4:**
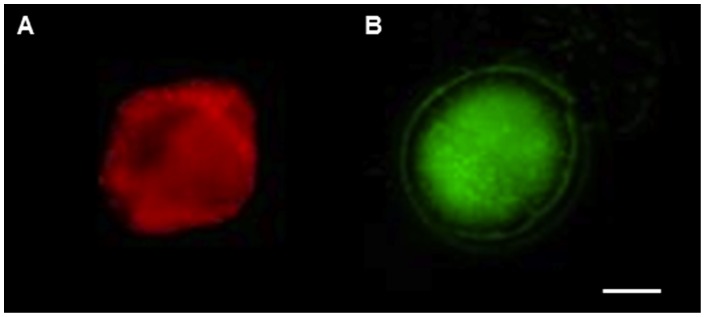
*Lingulodinium polyedrum* cells from sediment traps. Vegetative cells from sediment traps deployed on October 5, 2011. A. Vegetative cells with red chlorophyll autofluorencese (Ex. 450 nm, Em. 680 nm). B. Cyst cell with green autofluorecence (Ex.495 nm, Em.520 nm), objective 20X. Scale bar: 10 µm.

In the laboratory, a 250 ml subsample of the content of each trap was fixed with glutaraldehyde 1% final concentration to preserve red autofluorescene. Samples were sonicated for 5 minutes in a sonication bath to mix the sample and resuspend the cells. Then cells in suspension were counted in a 20 µl glass counting chamber using an epifluorescence microscope (Zeiss Axioskope 2 plus with a Xenon lamp) under blue light (450–490 nm excitation, dicroic 510 nm, >515 nm emission). Vegetative cells with red auto-fluorescence were easily distinguished from cyst with green auto-fluorescence (Figure 4). The vegetative migrating cells counted in the sediment traps were normalized to the cross section of the sediment trap (cell m^−2^) and then compared with the surface population during the day. To estimate the cells contained within the surface layer the in situ chlorophyll fluorometer signal was integrated from 0 to 3 m, and then converted to cell m^−2^ based on the comparison of cell abundance in Niskin samples and the in situ chlorophyll fluorescence at the corresponding depths (R^2^ = 0.7784, p<0.05). To determine the daily percentage of migrating cells the migrating vegetative cells in the sediment traps (cell m^−2^) were compared with the cells contained in the near surface water column integrated from 0 to 3 m depth (cell m^−2^).

## Results

### Near surface temperature stratification (NSTS)

**Figure 5:**
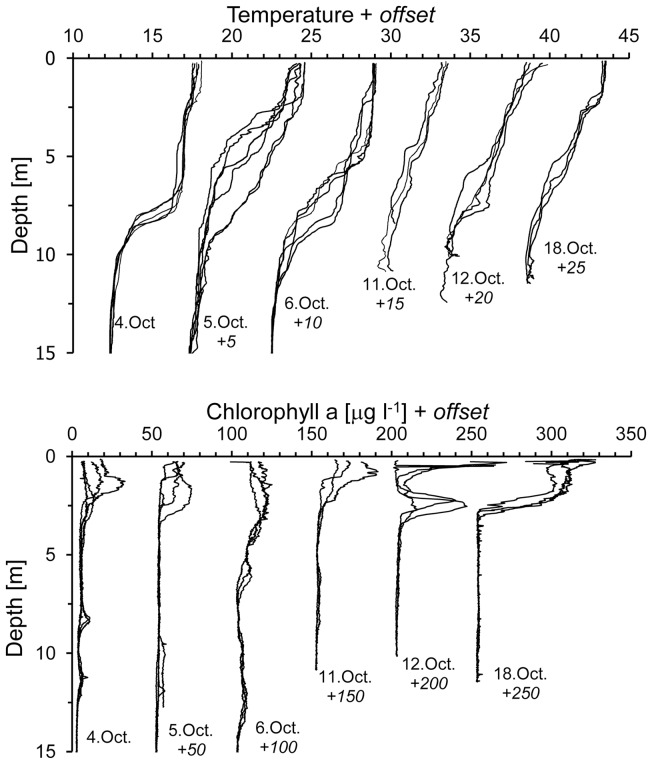
CTD profiles taken on October 4, 5, 6, 11, 12, and 18, 2011 during a dense algal bloom in Todos Santos Bay, Mexico. Temperature profiles (A) and chlorophyll profiles (B) Profiles of the different days are offset as indicated at the bottom below the dates.

Temperatures within the surface layer were logged by three thermographs attached to each of the drifters at 1, 3 and 5 m depths. Data analysis indicated no differences (p<0.05) between thermographs at the same depth but attached to different drifters, and no clear trend during deployment, suggesting little horizontal differences in temperature structure and no strong diurnal cycle in temperature. There were significant differences in temperatures between 1 to 3 m and 3 to 5 m (p<0.05) with temperature differences from 1.5 to 3.5°C over 2 m. Thermograph temperatures within the superficial layer were between 17.5 and 19°C for October 4, 5, 6, 11 and 12; but for the last sampling date (October 18) the temperature decreased to between 15.5 and 18°C. Temperature gradients did not increase during the day, implying that the temperature gradient was already present when the bloom organisms arrived at the surface and the drifters where deployed. Typically the CTD profiles showed a linear decrease in temperature with depth and no marked temperature step identifying a thermocline that would separate a surface layer from the rest of the water column, except for step changes in temperature observed on October 5 and 18 (Figure 5A). For our data we used a qualitative definition of thermocline because common characterizations are cumbersome to apply [31] and our depth scale is much smaller than typical oceanographic applications. Profiles that showed a marked near surface thermocline coincide with the lowest gradients registered between 1 and 3 m thermistors. On October 4 and 6 a thermocline at 8 m depth could be observed and some individual profiles of the other days showed weak step changes in temperature. The profiles show the inadequacy of defining a thermocline by a decrease of 0.8°C below the sea surface temperature [31]. In summary, data from thermistors did not show significant differences between gradient 1–3 m to the gradient 3–5 m as would be expected if a thermocline would be present above 5 m (Table 1); instead we typically observed a continuous thermal gradient for 1 to 5 m in most CTD profiles.

**Table 1:** Near surface temperature stratification, wind driven transport and vertical migration during a *Lingulodium polyedrum* bloom in 2011.

	NSTS		Surface transport	Surface bloom biomass			Cells reaching sea floor	
Date	ΔT(1–3m)	ΔT(3–5m)		Chl	Abundance	Lower depth limit		surface bloom
(dd:mm:yy)	[°C]	[°C]	[km]	[mg m^−2^]	[cell m^−2^]	[m]	[cell m^−2^]	%
04:10:2011	0.623	0.516	2.67	60	3.45E+09	2.7	nd	nd
05:10:2011	0.561	Nd	2.45	54	3.09E+09	2.8	nd	nd
06:10:2011	0.231	0.530	3.47	88	2.30E+09	3.3	1.28E+08	5.58
11:10:2011	0699	1.228	1.97	36	2.00E+09	1.96	nd	nd
12:10:2011	Nd	Nd	2.93	55	3.9E+09	2.98	6.47E+07	1.66
18:10:2011	0.399	2.252	2.7	138	6.7E+09	2.8	1.81E+08	2.7

NSTS is the near surface temperature stratification given as temperature difference within 2 m depth intervals. The surface transport is calculated as the excursion for 8 hours of onshore wind. Surface bloom biomass is the integral from the surface to the ‘lower depth limit’. Cells reaching the seafloor by vertical migration are collected in the traps during night and given in cells per area and in percentage of surface bloom biomass. No data is specified by nd.

### Bloom forming organism, the thermal layer and surface bloom wind drift (SBWD)

*In situ* fluorescence profiles were used to document the presence of the bloom forming organisms within the NSTS. Highest values of chlorophyll concentration were found on October 12 and 18, 2011 (Figure 5B). On October 12 the concentration was 138 mg m^−2^ integrated from 0 to 3 m. On October 4, 5, 6 and 11 the chlorophyll concentration reached 88 mg m^−2^ between 0 and 3 m. In summary, chlorophyll profiles during the sampling showed a heterogenous distribution and cells were concentrated within thin layers.

**Figure 6:**
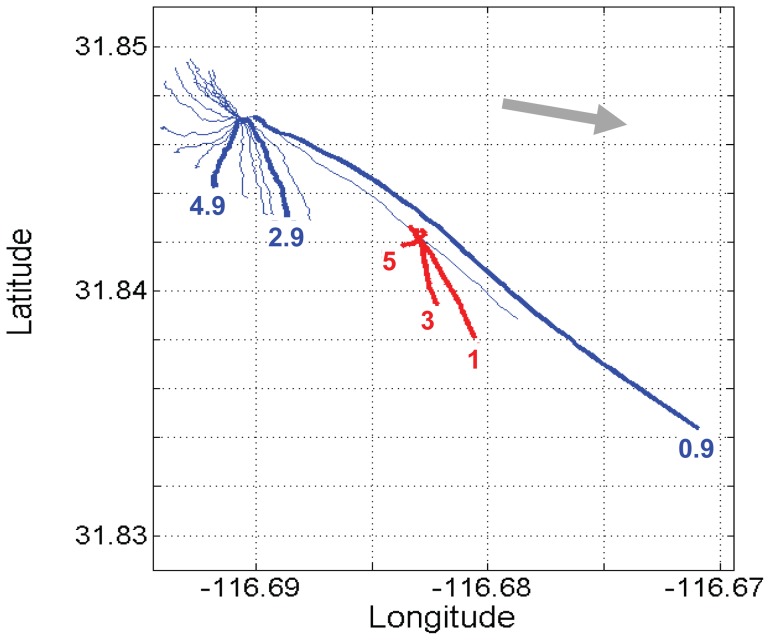
Virtual displacement from ADCP (Aquadopp, Nortek) and CODE-type drifter trajectories on September 21, 2011. Depths are indicated at the end of the trajectories. ADCP (blue), Drifters (red). ADCP trajectories are for each 0.5 m interval. The dominant wind direction is indicated by the grey arrow.

**Figure 7:**
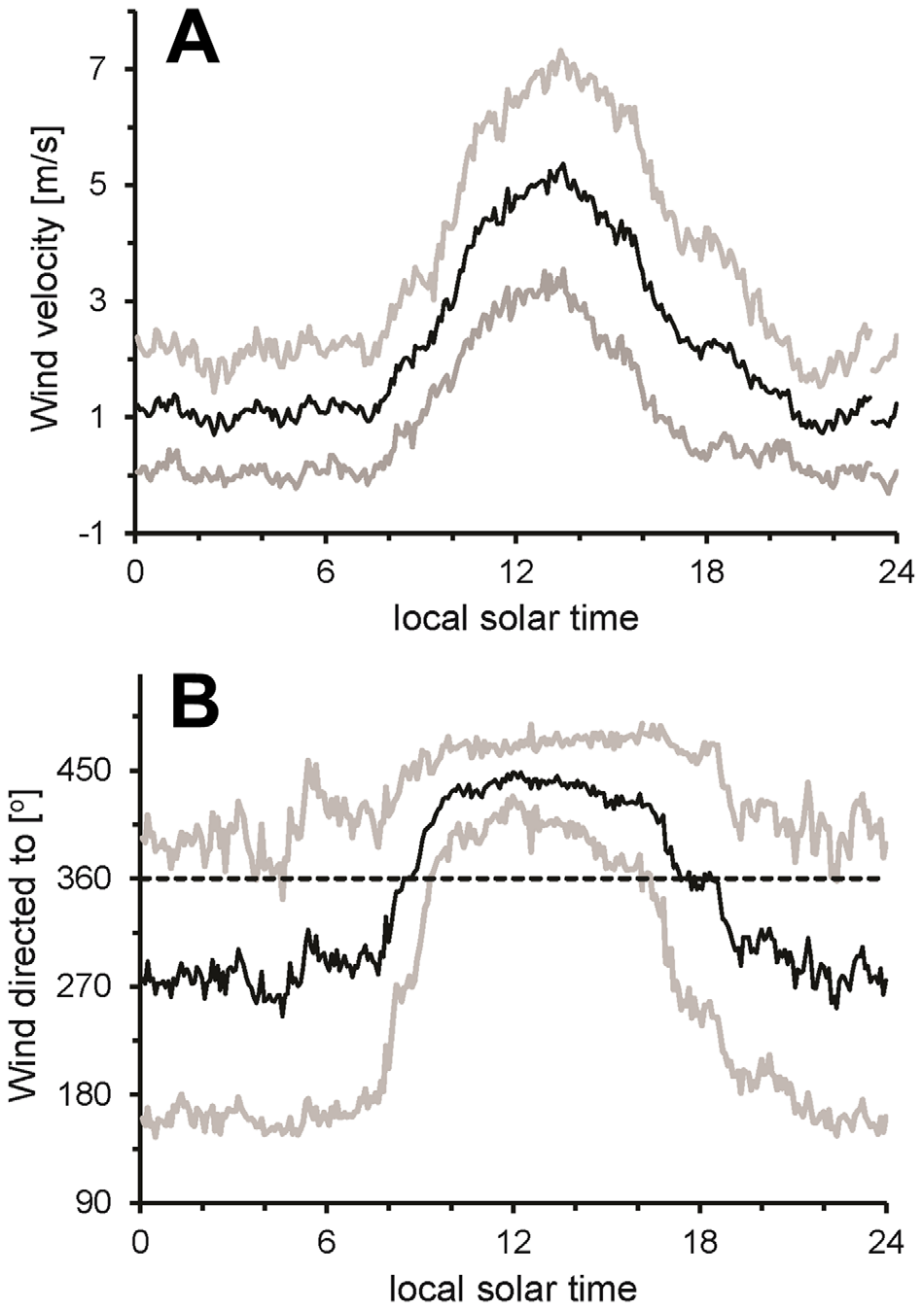
Wind pattern during October 2011. Average values (black) +/− standard deviation (grey). 12∶00 of local solar time represent the time of minimum zenith angle. (A) Wind direction and (B) Wind speed [m s^−1^].

Surface coastal circulation can be followed by tracking the position of drifters which are expected to stay in the same parcel of water, however additional external forces can act on the drifters such as wind resistance on the surface buoy or currents acting on the cable suspending the drogue. CODE drifters with drogue bodies close to the surface can be affected by the water motion induced by surface waves, thus changing the direction and speed of the drifter from the net horizontal transport. In the methodology section, we confirmed the water-following capability of CODE drifters at 1 and 5 m depth by comparing velocities and bearing with Holey sock drifters. We also validated the CODE drifters with an ADCP, finding on September 21, 28 and 29 (2011) similar trajectories and velocities as the trajectories and velocities of 1, 3 and 5 meters CODE drifter. During the three days, ADCP trajectories showed a spiral current vector distribution with depth where the surface current follows approximately the wind direction and lower current vectors represent local hydrographic patterns (Figure 6). The similarity in surface current and wind is the result of the uncoupling of the water close to the surface from deeper water currents made possible by the near surface stratification. In general, data obtained from these comparisons suggest that drifter-derived velocities (Lagrangian velocity) do agree with the velocities measured by the ADCP (Eulerian velocity). During ADCP deployment wind conditions were different from bloom conditions: the wind was directed to 110 degrees with average wind velocities of 0.33 m s^−1^ for the three days of ADCP deployments (Figure 6). The wind speed was significantly lower than the mean velocity registered during bloom conditions in October 2011 (approximately 5 m s^−1^) (Figure 7) and we cannot exclude that the ADCP-drifter comparison would have resulted less similar with higher wind velocities.

### Wind driven transport and drifters trajectories

**Figure 8:**
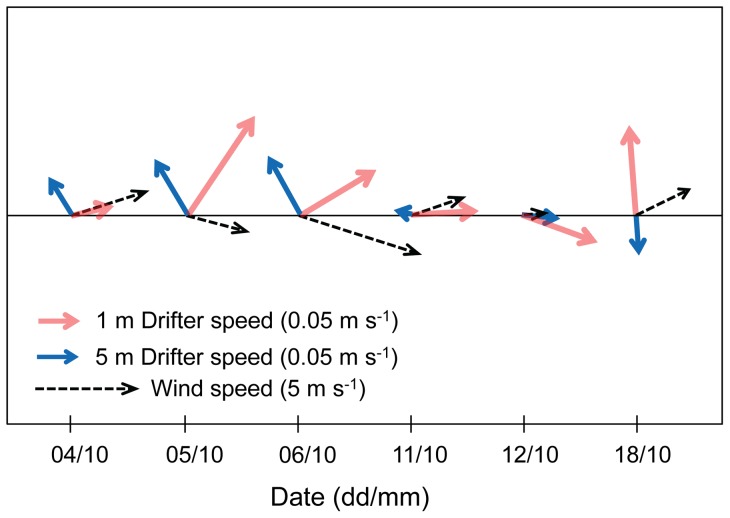
Drifter and wind vectors. Averages are from 13 to 15 hrs on October 4, 5, 6, 11, 12 and 18, 2011. Drifter velocities (1 m, red; 5m, blue) differ in scale from wind velocities (broken black). North is in the ordinate direction, and East in the abscissa.

Monthly wind pattern on October, 2011 showed a diurnal breeze pattern with increasing velocities (Figure 7A) and wind directed toward the coast between 9 to 17 hrs (Figure 7B), resulting in eight hours of thermal breeze, with an average of 5±2 m s^−1^ at midday. We compared the period with the highest velocities between 13 to 15 hrs with the trajectories of 1 and 5 meters drifters (Figure 8). In general, the wind speed measured during the period of 13 to 15 hrs, were from 1 to 6 m s^−1^ and wind direction was directed toward the coast (Figure 8); drifters were transported at speed of up to 0.05 m s^−1^ during the sampling dates. The 1 m drifter directions were toward the coast except for October 18^th^, these drifters trajectories were obtained from Holey sock drifter data. The 5 m drifters showed direction very different from the 1 m drifters mostly away from the coast and showed velocities between 0.004 and 0.07 m s^−1^ generally smaller than 1 m drifters. The time course of wind and surface drifter vectors showed oscillations of approximately 20 min periods. These oscillations are probably the result of Seiches within the bay and the period corresponds to the approximate dimensions of the bay with a depth of 20 m and a diameter of 10 km. In the video (Video S1) we are showing the drifter trajectories for two days, with the 1 m drifter generally more directed towards the coast whereas the 5 m drifter trajectory pointed away. The 3 m drifter had trajectories between the 1 and 5 m drifters. Some of the drifters showed a change in direction during deployment which could be the result of a delayed response to wind forcing or due to tidal currents.

The general information about NSTS, wind driven transport, and vertical migration documented in this work are summarize in Table 1. We estimated the distance traveled by surface buoys during the eight hours of thermal breeze for each sampling day; superficial water parcel traveled on average 2.7 km each day. On October 4, 5 and 6 superficial drifters moved 794, 1058, and 1190 m in 2.8, 3.45, and 2.75 hours respectively. On the second week of the sampling for October 11 and 12 the drifters moved 675 and 1264 m in 2.75 and 3.45 hours respectively. On October 18, the GPS attached on the 1 m drifter turned off during the sampling, so we could not calculate the final transport.

### *L. polyedrum* cells in sediment traps

In the traps mostly vegetative cells were found, they could be identified by their red chlorophyll autofluorescence by epifluorescence microscopy and also by cell shape (Figure 4). Green auto-fluorecence (GAF) intensity increases with time after cell death or fixation and with excitation by blue or UV light [32]. We found GAF could help to easily discrimate cysts from vegetative cells (Figure 4). In this paper, we present cells abundance found inside the traps for each day of traps deployement and its proportion compared with the integrated biomass present in the surface layer (0 to 3 m). For the three days of sediment trap cell counts the samples showed that around 3% of the surface population reached the sediment (Table 1).

## Discussion and Conclusions

Surface blooms in coastal waters have socioeconomic impacts reaching from respiratory health issues to aquaculture commerce and tourist perceptions [33], [34], [2]. Assuming that near surface thermal stratification is a common occurrence, the transport mechanism discussed here helps explain surface bloom dynamics in many parts of the world where sea breezes are commonly present [20]. Unfortunately few trustworthy data on near surface stratification exist because when regular profiling instruments are deployed next to research platforms the data would be subject to a likely but unknown mixing effect. Near surface stratification has been shown in Mexican Pacific waters using specialized instrumentation [35], [36] and other studies about near surface stratification are based on shipboard radiometric measurements applied to a physics-based model of near-surface warming [37]. By contrast, there is extensive literature on the interpretation of sea surface temperature (SST) because of the possibility of remote sensing, but SST probably has a different dynamic from NSTS. The diurnal SST cycle with cooling during the night has been observed and related to the near surface stratification but more in situ information on near surface stratification is lacking [38], [39], [40]. It is therefore for the first time that we show near surface stratification and wind-driven transport of the surface layer.

Our initial hypothesis considered that the high daylight attenuation in surface blooms would help the formation of the NSTS, however we did not observe any bloom-related surface layer heating, which indicates that NSTS is independent from the formation of the blooms. Approximately half of daylight energy is contained in the infrared radiation (IRR) and is being absorbed within the first cm of the water independent of the phytoplankton concentration. Near IRR (<1000 nm) reflectance is slightly higher and therefore IR absorption slightly reduced in blooms because of high particle back scattering at the surface but we consider this of minor importance to the near surface temperature budget [41]. The visible light is highly attenuated near the surface in blooms; the PAR profiles of the free-rising CTD obtained within the high chlorophyll patches showed very high PAR attenuation, PAR levels at 2 m depth were between 1 and 10 percent of surface PAR when the profiles were extrapolated to 0 meters depth (data not shown). Despite this high attenuation of PAR near the surface, *in situ* temperature measurements during periods of high PAR levels showed no evidence of increased heating of surface waters within blooms when surface temperatures inside and outside bloom patches were compared (data not shown). Also NSTS has been observed in a coastal station without bloom conditions outside the bay (data not showed).

Generally NSTS took the shape of a continuous gradient and typically showed no marked discontinuity that would indicate a homogenous near surface layer. In Figure 5A temperature steps near 2.5 m depth were found on October 5 and 18 but in most cases these steps were absent. The predominantly continuous gradient was contrary to our initial concept (Figure 1), since we hypothesized the formation of a diurnal near surface thermocline with a thin homogeneous surface layer. The NSTS reported here showed no change in stratification pattern during the sampling period, but we sampled only during the day when blooms could be observed at the surface, and therefore we have no information on a possible diurnal patters as described before [42].

The wind stress at the sea surface is transmitted down the water column, but because of the continuous stratification the resulting shear flow produced a spiral of current vectors over a small depth range of less than 10 m. This shear flow spiral can turn in either direction depending on the azimuth relation of wind stress and deeper water current. The shear flow spiral is also present in the ADCP data taken from 10 m upwards before the bloom period (Figure 6). The drifter trajectories during the bloom followed a similar spiral pattern as seen in the video (Video S1). Since this flow spiral shows as the sudden response of varying winds, it bears no resemblance to Ekman-like spirals which are the steady end conditions. In contrast to the Ekman spiral the shear flow can turn either way without diminishing with depth [43]. In some instances step changes in the temperature profiles were found (October 5 and 18), under those conditions the spiral shear flow pattern might change but our drifter data do not indicate that. In the vicinity of 30° latitude, breezes are near resonant with inertial waves, which, even with the lack of lateral freedom imposed by the coast, generate relatively large, daily-oscillating ocean movements. It has been proposed that these daily oscillations favor nutrient supply towards the surface and the coast [20], [44]. For the purpose of ecology bloom transport, we consider here only the onshore transport due to wind (Figure 7A and B). The relationship between wind pattern and water movement is observed in Figure 8, wind data indicate onshore trajectories with maximum velocities of 5.1 m s^−1^, a typical velocity reported for this location [45]. During the day the strong winds associated with the daily sea breeze can produce onshore surface currents at 1 m depth with velocities of up to 0.01 m s^−1^ similar to the 0.01 m s^−1^ to 0.015 m s^−1^ found near our site during summer conditions [46], [17].

**Figure 9:**
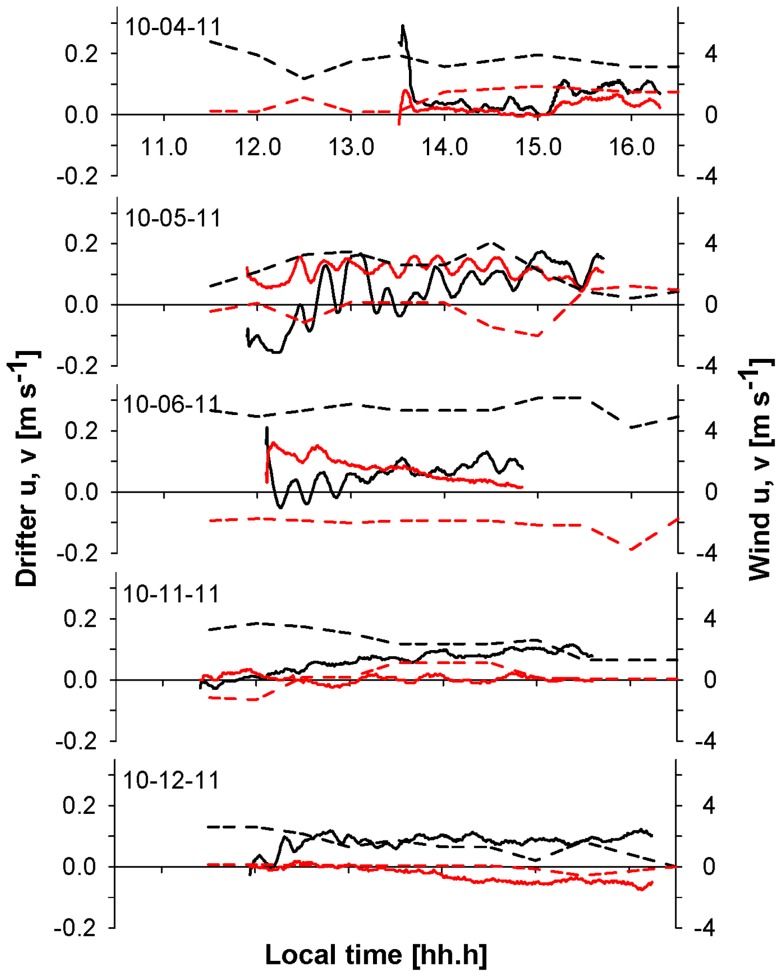
1m drifter and wind velocity components. Time series of the longitudinal (u) and latitudinal (v) components, with scales as indicated on ordinates. Wind (dashed lines), drifters (solid lines), longitudinal u (black), latitudinal v (red).

The general pattern of drifter trajectories for all days showed differences between surface and 5 m drifter trajectories (Figure 8). On October 5, 6 and 18, surface drifters did not have the same direction as the wind, which probably resulted from an interaction between wind and current forcing. A similar wind-current interaction has been observed during a tracer transport experiment at less than 1 m depth inducing larvae on-shore transport by sea breeze [16], [17]. Oscillations in vector direction and velocity with periods of approximately 20 min [47] have been interpreted as Seiches (Figure 9), however in this study these oscillations were not considered to have a significant effect on the overall transport of the water in the top 3 meter.

### Ecological implications of the SBWD

The combination of diel vertical migration and diurnal onshore transport facilitates the maintenance of bloom forming organisms. Surface bloom forming dinoflagellates show a typical diel migration pattern that allows the population to obtain nutrients during night and photosynthesize during the day at the surface [25], [48], [49]. When sufficient nutrients are available at the surface, the dinoflagellates show reduced vertical migration tendencies [22]. Vertical migration also provides *L. polyedrum* at night with a less turbulent environment during the time of cell division, potentially beneficial for *L. polyedrum* because of its sensitivity to shear flow during cell division [50]. Arguments linking nutrient limitation and vertical migration have considered inorganic nutrients but not vitamins. *L. polyedrum* is vitamin B12 auxotroph [51], maybe the benthic bacteria provide them with the necessary vitamin B12 for population growth? There are still many ecophysiological aspects of bloom development unknown. Here we argue that the shoreward transport of the bloom helps to maintain the bloom and increases the possible redevelopment of the bloom in the future, considering the arguments provided by the literature: Bloom organisms reaching the sediment would have more nutrients available [27], avoid horizontally dispersion during the night [52], and have shallower seedbeds for the initiation of future blooms [53], [54].

**Figure 10:**
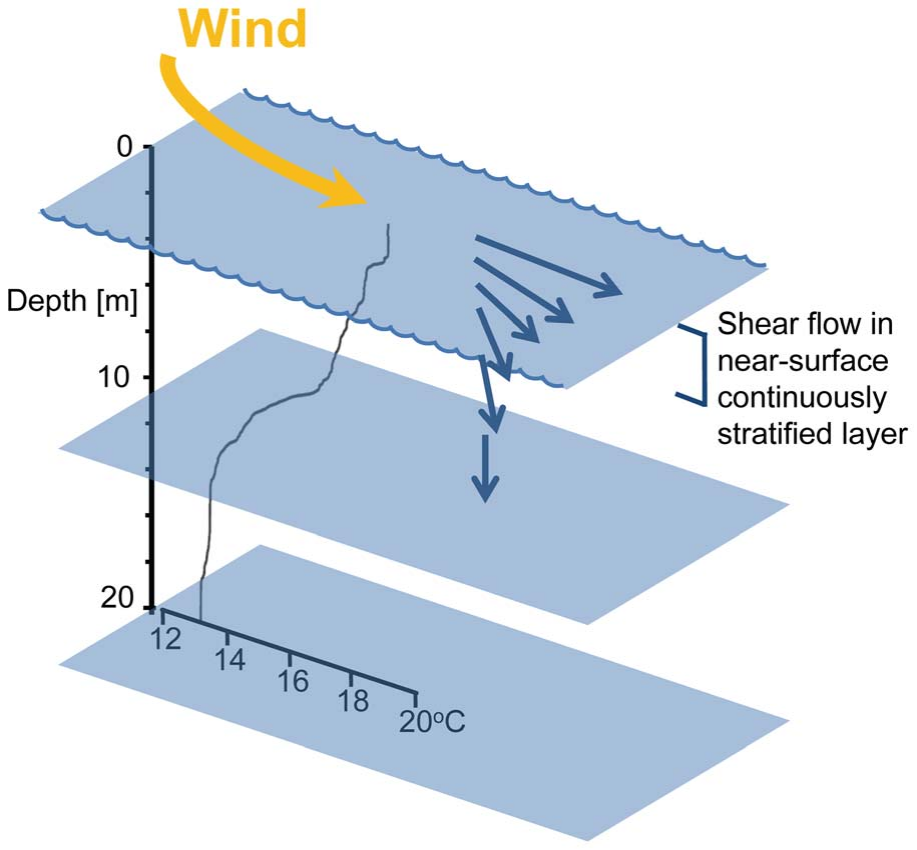
Schematic representation of continuous temperature stratification in the first meters and near surface shear flow. Temperature profile from 12-Oct-2011, current vectors are hypothetical.

We found high cell densities of *L. polyedrum* in near surface thin layers (Figure 5, 12-Oct-2011). Different mechanisms forming thin layers have been proposed, from physical processes, such as the interaction between vertical shear gradients and the horizontal advection of phytoplankton patches to physiological adaptations that increase the net growth rate [55], [10], [6], [56], [57]. We propose an additional mechanism based on the shear flow spiral and the patchy horizontal distribution of the bloom: The spiral shows current vectors of small vertical distance with significantly different azimuth directions (Figure 10). A bloom patch that is vertically homogenous over the top 3 meters can be transported in different directions and a deeper layer can be transported into a region with no bloom at the surface. This transport could lead to the observed thin layer (Figure 5, 12-Oct-2011) but it could also help to distribute the bloom patches horizontally and dilute the concentration in bloom patches assuming that the organisms would migrate to vertically redistribute themselves in the surface layer.

We observed a net onshore transport of blooms of approximately 2.7 km day^−1^ calculated from the average drifter velocity and the period of coast wise sea breeze. A cell with an average generation time of two days [21] would be transported 5.4 km onshore before cell division. This distance should be significant considering that extensive *L. polyedrum* blooms have been observed near the coast of California, typically found between 500 m [58] to less than 20 km from shore, for example near our coast during a dense algal bloom in 2005. The onshore movement becomes important because it coincides with nutrient gradients perpendicular to the coast over this distance. Also the sea floor depth will be shallower making it easier to be reached during down migration. Our sediment trap data were placed below high concentration surface patches and collected about three percent of the vegetative surface population during one night, accompanied by a low number of cysts; the percentage of migrating cells would be higher if calculated using the average surface *L. polyedrum* concentration in the bay. Considering a maximum swimming speed of 278 µm s^−1^ for *L. polyedrum* [59], [60] the surface population can reach 12 m depth within 12 hours, a depth close to the deployment depth of the traps. These deep migrating cells might not migrate to the surface the same day. Cysts can reach the sediment by sinking passively through the water column during the night. Cysts that reach the sediment at a shallower depth could presumably return to the vegetative state and start a new bloom under more favorable conditions. The relationship between the cell cycle and diurnal vertical migration has been recognized as an important part of bloom dynamics [61]; however, swimming behavior may vary among dinoflagellate species [62] and under different environmental conditions such as temperature stratification, light [63], [64] and nutrient limitation [65], [23], [66]. In either case, a more shallow water environment will be beneficial to grow, divide, or leave the cysts for the next bloom.

In the present study, we demonstrated the presence of near surface stratification that is promoting the wind drift of the surface layer. We showed how this wind drift could transport surface blooms closer to the coast and we suggest that this mechanism is helping the organisms to maintain the bloom. However, wind drift of the surface water will not only transport blooms, but also all dissolved and particulate constituents of the surface water; some of these constituents could be larvae, or contaminants released near the coast with ship ballast water or from waste water outflows. Water contaminants, as well as surface bloom in coastal waters have socioeconomic impacts reaching from respiratory heath issues to aquaculture damage and the interference with tourism [32], [33], [2]. This transport has been rarely documented in coastal waters and its consequences have been little considered in the literature.

## Supporting Information

Video S1 **Drifter trajectories off Ensenada, Baja California (31.855° N, 116.665° W) during a dense surface algae bloom on October 5 and 6, 2011.** Drifter colors indicate drogue depth: red 1 m, green 3 m, yellow 5 m. The on-shore red arrow indicates wind direction and strength (red), scale bars for geographic distance (black) and wind strength (red), and shore line (black) are included.
(AVI)
