# Correction: Maintenance of Coastal Surface Blooms by Surface Temperature Stratification and Wind Drift

**DOI:** 10.1371/annotation/a2f49bbd-e226-4a15-900a-5946cff07d75

**Published:** 2013-06-12

**Authors:** Mary Carmen Ruiz-de la Torre, Helmut Maske, José Ochoa, César O. Almeda-Jauregui

The following information is missing from the Funding Statement:

The Mexican CONACyT funded this project (CB--2008-01 106003) and provided the doctoral fellowship to MC Ruiz de la Torre. 

